# Correction: Tokusanai et al. *Prunus mume* Extract Inhibits SARS-CoV-2 and Influenza Virus Infection In Vitro by Directly Targeting Viral Particles. *Int. J. Mol. Sci.* 2025, *26*, 8487

**DOI:** 10.3390/ijms27020625

**Published:** 2026-01-08

**Authors:** Mizuki Tokusanai, Koichiro Tateishi, Kanako Hirata, Nahoko Fukunishi, Yusuke Suzuki, Ryohei Kono, Sorama Natsumi, Chikara Kato, Susumu Takekoshi, Yoshiharu Okuno, Hirotoshi Utsunomiya, Norio Yamamoto

**Affiliations:** 1 Department of Microbiology, Tokai University School of Medicine, 143 Shimokasuya, Isehara 259-1193, Kanagawa, Japan; 2cmud015@mail.u-tokai.ac.jp (M.T.); tateishi.koichiro.f@tokai.ac.jp (K.T.); 3cmud009@mail.u-tokai.ac.jp (K.H.); katou.chikara.n@tokai.ac.jp (C.K.); susumu.takekoshi@nifty.ne.jp (S.T.); 2 Department of Emergency and Critical Care Medicine, Tokai University School of Medicine, 143 Shimokasuya, Isehara 259-1193, Kanagawa, Japan; 3 Department of Life Science Support, Research Innovation Center, University Hospitals Sector, Tokai University, 143 Shimokasuya, Isehara 259-1193, Kanagawa, Japan; n-fuku@tokai.ac.jp (N.F.); s.yusuke0220@tokai.ac.jp (Y.S.); 4 Department of Rehabilitation, Osaka Kawasaki Rehabilitation University, 158 Mizuma, Kaizuka 597-0104, Osaka, Japan; konor@kawasakigakuen.ac.jp (R.K.); utsu@kawasakigakuen.ac.jp (H.U.); 5 Department of Applied Chemistry and Biochemistry, National Institute of Technology, Wakayama College, 77 Noshima, Nada, Gobo 644-0023, Wakayama, Japan; natsumi-sorama@i.softbank.jp (S.N.); okuno@wakayama-nct.ac.jp (Y.O.)

In the original publication [1], an error was identified in Figure 5B. The graph for PM2/Ancestral was inadvertently duplicated and used in place of the PM2/Delta panel. As a result, two PM2/Ancestral graphs were presented, and the PM2/Delta graph was omitted. The corrected Figure 5B appears below. The authors state that the scientific conclusions are unaffected. This correction was approved by the Academic Editor. The original publication has also been updated.

## Figures and Tables

**Figure 5:**
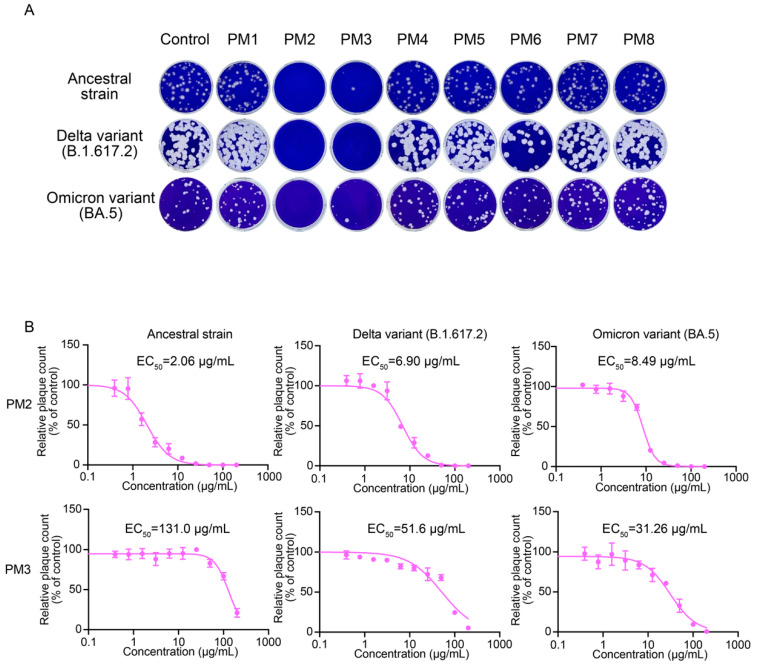
Antiviral effects of *P. mume* extracts against SARS-CoV-2 evaluated by plaque assay. (**A**) Representative plaque assay images showing SARS-CoV-2 treated with PM1–PM8 at a 200 µg/mL concentration. (**B**) EC_50_ values of PM2 and PM3 were determined by plaque assays. Plaques were counted, and relative plaque counts were calculated as percentages of the untreated virus control. Data are presented as the mean ± SD from three independent experiments. EC_50_ values were determined by a four-parameter logistic model.
